# Shwachman-Diamond Syndrome: frequent misdiagnosis as Jeune Syndrome and other peculiarities

**DOI:** 10.1186/1546-0096-9-S1-P313

**Published:** 2011-09-14

**Authors:** I Meyts, H Schaballie, F Haerynck, L Sevenants, C Vermylen, V Bordon, X Bossuyt, A Corveleyn, A Uyttebroeck, M Renard

**Affiliations:** 1 Department of Pediatric Immune Deficiencies, University Hospitals Leuven, Belgium; 2 Department of Pediatric Pulmonology and Immune Deficiencies, University Hospital Ghent, Belgium; 3 Department of Pediatric Hemato-Oncology, Cliniques Universitaires St Luc, Woluwe, Belgium; 4 Department of Pediatric Hemato-Oncology, University Hospital Ghent, Belgium; 5 Department of Laboratory Medicine, University Hospitals Leuven, Belgium; 6 Center for Medical Genetics, University Hospitals Leuven, Belgium; 7 Department of Pediatric Hemato-Oncology, University Hospitals Leuven, Belgium

## Background

Shwachman-Diamond Syndrome (SDS) is rare inherited disorder. The typical diagnostic triad (neutropenia, skeletal dysplasia and exocrine pancreatic insufficiency) is not always present at diagnosis.

## Aims

To review mutations and initial presentation in a Belgian cohort of patients with genetically proven Shwachman-Diamond Syndrome (SDS).

## Methods

A retrospective study in 11 patients with SBDS mutations.

## Results

In 10 patients an SBDS mutation was identified in both alleles, patient 11 was heterozygous. The mean age at diagnosis was 2.9 years. All patients had exocrine pancreatic insufficiency. Radiological evidence of skeletal dysplasia was present in 9/10 studied. Neutropenia was present in 8/11 patients. Failure to thrive was demonstrated for all but P8. 2/3 patients experiencing cholestatic hepatitis required admission to ICU. Both had blood CMV PCR(+). The 3^rd^ patient suffers from chronic liver failure due to liver fibrosis. 10/11 experienced recurrent infections (septicemia, respiratory tract infections, skin infections). Two patients had an episode of symptomatic (convulsions) hypoglycemia without satisfying explanation despite extensive metabolic analysis.

3/11 patients received a diagnosis of Jeune syndrome (one patient died of respiratory insufficiency) and 1/11 of hypobetalipoproteinemia prior to diagnosis of SDS. A metabolic disorder was first suspected in P11 because of hypertrophic cardiomyopathy. Two couples of siblings in our cohort showed an entirely different course.

## Conclusion

SDS triad was present at diagnosis in only 6/9. A high index of suspicion is crucial. The peculiar misdiagnoses as Jeune syndrome is striking as are the episodes of symptomatic hypoglycemia and the suspected increased susceptibility to severe CMV disease.

